# Evaluation of Antimicrobial Efficiency of New Polymers Comprised by Covalently Attached and/or Electrostatically Bound Bacteriostatic Species, Based on Quaternary Ammonium Compounds

**DOI:** 10.3390/molecules201219768

**Published:** 2015-12-01

**Authors:** Efstathia Kougia, Maria Tselepi, Gavriil Vasilopoulos, Georgia Ch. Lainioti, Nikos D. Koromilas, Denisa Druvari, Georgios Bokias, Apostolos Vantarakis, Joannis K. Kallitsis

**Affiliations:** 1 Environmental Microbiology, Department of Public Health, Medical School, University of Patras, 26504 Patras, Greece; efi.84@hotmail.com (E.K.); mrtselepi@gmail.com (M.T.); gavriilvasilopoulos@gmail.com (G.V.); 2 Department of Chemistry, University of Patras, 26504 Patras, Greece; glainioti@upatras.gr (G.C.L.); nikoskoromil@upatras.gr (N.D.K.); druvari@upatras.gr (D.D.); bokias@upatras.gr (G.B.)

**Keywords:** antimicrobial activity, polymers, quaternary ammonium units, covalent attachment, electrostatic binding, bacteria, survival

## Abstract

In the present work a detailed study of new bacteriostatic copolymers with quaternized ammonium groups introduced in the polymer chain through covalent attachment or electrostatic interaction, was performed. Different copolymers have been considered since beside the active species, the hydrophobic/hydrophilic nature of the co-monomer was also evaluated in the case of covalently attached bacteriostatic groups, aiming at achieving permanent antibacterial activity. Homopolymers with quaternized ammonium/phosphonium groups were also tested for comparison reasons. The antimicrobial activity of the synthesized polymers after 3 and 24 h of exposure at 4 and 22 °C was investigated on cultures of Gram-negative (*P. aeruginosa*, *E. coli*) and Gram-positive (*S. aureus*, *E. faecalis*) bacteria. It was found that the combination of the hydrophilic monomer acrylic acid (AA), at low contents, with the covalently attached bacteriostatic group vinyl benzyl dimethylhexadecylammonium chloride (VBCHAM) in the copolymer P(AA-co-VBCHAM88), resulted in a high bacteriostatic activity against *P. aeruginosa* and *E. faecalis* (6 log reduction in certain cases). Moreover, the combination of covalently attached VBCHAM units with electrostatically bound cetyltrimethylammonium 4-styrene sulfonate (SSAmC_16_) units in the P(SSAmC_16_-co-VBCHAMx) copolymers led to efficient antimicrobial materials, especially against Gram-positive bacteria, where a log reduction between 4.9 and 6.2 was verified. These materials remain remarkably efficient even when they are incorporated in polysulfone membranes.

## 1. Introduction

The development of polymers with antimicrobial properties is an important area of research focused on different fields of applications, like hospital environments (surfaces/furniture), surgery equipment, water purification systems, food packaging and storage, antifouling paints, *etc.* Infections, caused by pathogenic microorganisms, due to contact with solid surfaces, is an emerging and well-known problem that has captured the attention of the scientific community in the last years [1]. Great efforts have been made, especially in hospitals, to overcome this problem [2,3]. Numerous epidemiological studies have shown that the most common bacteria causing hospital infections are *Pseudomonas aeruginosa*, *Staphylococcus aureus*, *Enterococci* and *Escherichia coli* [4,5,6,7]. Multi-resistant bacteria such as methicillin-resistant *Staphylococcus aureus* (MRSA) [8] or vancomycin-resistant *Enterococci* (VRE) [9] are of great concern in hospital environments and continue to challenge infection control and epidemiology practice worldwide.

One mechanism of action of low molecular weight antimicrobial compounds, is to affect the adhesion of bacteria by reducing their contact ability to the surface (without killing them), which may result in environmental contamination and toxicity to the human body due to bacteriostatic diffusion [10]. Alternatively, polymers with antimicrobial units such as quaternary ammonium or phosphonium salts, guanides, peptides and antibiotics commonly kill bacteria on contact [11,12,13]. In addition, they ensure chemical stability, non-volatility, as well as long-term activity. The mechanism of the bacteriostatic action of the polycationic biocides involves a destructive interaction with the cell wall and/or cytoplasmic membranes [14]. Quaternary ammonium or phosphonium compounds (QAC or QPC) are cationic biocides that target the bacterial membranes. Polymers with quaternary ammonium or phosphonium salts are widely explored materials with potent antimicrobial activity [15,16,17,18] and effectiveness even against bacteria that are resistant to other cationic antibacterial agents [19]. Through either direct polymerization of monomers containing quaternary groups or incorporation of the quaternary moieties within the synthesized polymers, the final polymeric materials may have potential antimicrobial activity due to the intrinsic properties of the quaternary groups [20,21]. Depending on the mode of incorporation of QAC or QPC in the polymers, they can be classified in two categories: ionically bound or covalently attached [14,22,23,24]. The polymeric materials of the first class usually exhibit strong bacteriostatic action, based on the release of the active cations (QAC or QPC) in the aqueous environment through an ion exchange mechanism. On the other hand, the bacteriostatic action of the second class is based on the contact of the bacteriostatic polymer with the microorganisms.

The main goal of the present work was the evaluation of the bacteriostatic activity of both classes of novel polycationic biocides. Although QPC-containing polymers were also investigated, the present study is mostly focused on QAC-containing bacteriostatic polymers. The attachment of the quaternary ammonium groups onto the final polymeric material took place either covalently or electrostatically, or even through the combination of both methods. Moreover, in the case of copolymers with covalently attached biocides, the influence of the co-monomer nature (hydrophilic/hydrophobic/weakly acidic) was also explored. The antibacterial effects of the polymers were investigated on *in vitro* cultures of two Gram-negative (*P. aeruginosa* and *E. coli*) and two Gram-positive (*S. aureus* and *E. faecalis*) bacteria by testing the survival rate at different contact times (3 and 24 h) and temperatures (4 and 22 °C).

## 2. Results and Discussion 

### 2.1. Synthesis and Characterization of Polymers

**Figure 1:**
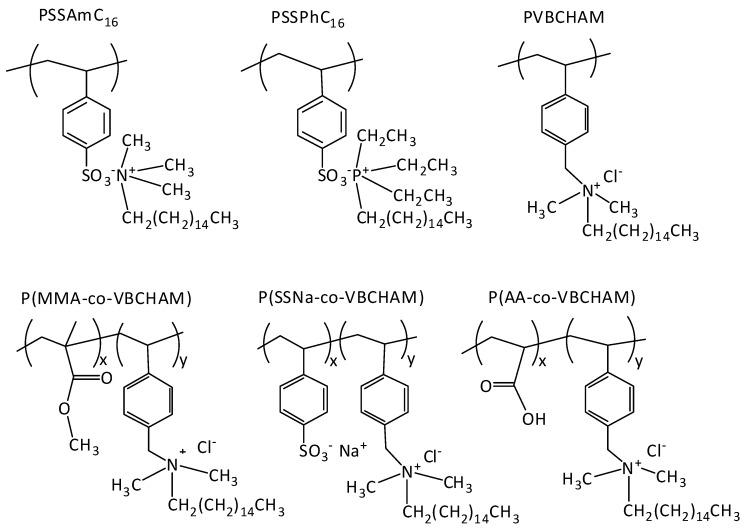
Chemical structures of the homopolymers PSSAmC_16_, PSSPhC_16_, PVBCHAM and the copolymers P(MMA-co-VBCHAM), P(SSNa-co-VBCHAM), P(AA-co-VBCHAM).

Quaternary ammonium and phosphonium salts are important biocides known to be effective against a broad spectrum of micro-organisms. As their antimicrobial efficacy depends to a great extent on the length of the alkyl chain [25], salts with long (hexadecyl) chains were evaluated in the present study. The chemical structure of the polymeric biocides under investigation is shown in Figure 1, while the nomenclature used and a brief description of their main characteristics and their chemical composition are presented in Table 1. As seen, the selected copolymers bear groups with potential antibacterial functionalities, such as quaternary ammonium groups, bound covalently or electrostatically onto the polymer chain. Thus, targeting a permanent antimicrobial behaviour in the case of covalent attachment of ammonium groups, different copolymers, bearing the active group vinyl benzyl dimethylhexadecylammonium chloride VBCHAM were studied, together with hydrophobic co-monomers, such as methyl methacrylate (P(MMA-co-VBCHAM47)-D5) or hydrophilic ones, such as sodium 4-styrene sulfonate (P(SSNa-co-VBCHAM20)-D6, P(SSNa-co-VBCHAM85)-D6a) and acrylic acid (P(AA-co-VBCHAM88)-D7, P(AA-co-VBCHAM20)-D7a). The bacteriostatic activity of these copolymers against a broad range of bacteria was compared to that of the homopolymers bearing quaternary ammonium units (PSSAmC_16_-D1) or quaternary phosphonium units (PSSPhC_16_-D4) as counter ions. The synthesis and characterization of these (co)polymers have been described in previous works [22,26].

**Scheme 1:**
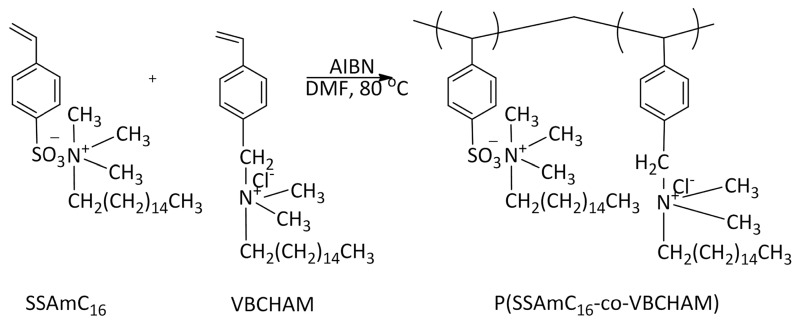
Reaction steps for the synthesis of the P(SSAmC_16_-co-VBCHAMx) copolymers.

In an attempt to combine the properties of both classes of bacteriostatic polymers, bearing electrostatically bound and covalently attached bacteriostatic units, the monomers SSAmC_16_ (with quaternary ammonium counter ions) and VBCHAM (with covalently attached quaternized nitrogen atoms) were synthesized (Scheme 1). These monomers were subsequently copolymerized through free radical polymerization (FRP), providing copolymers like P(SSAmC_16_-co-VBCHAM65)-D3 and P(SSAmC_16_-co-VBCHAM25)-D3a. For comparison reasons, the respective homopolymers, PSSAmC_16_-D1 and PVBCHAM-D2, were also studied.

**Figure 2:**
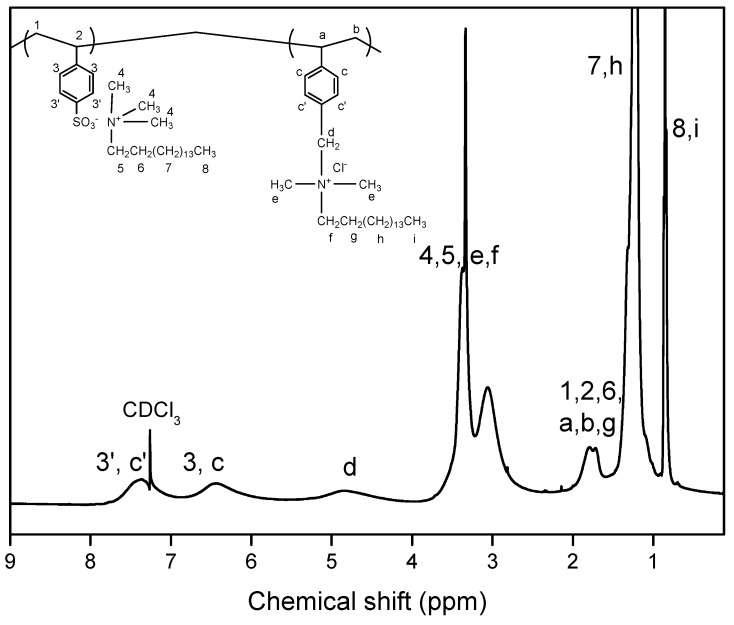
^1^H-NMR spectrum of P(SSAmC_16_-co-VBCHAM65).

The copolymers’ structure and content was verified through ^1^H-NMR spectroscopy. As an example, the ^1^H-NMR spectrum of the copolymer P(SSAmC_16_-co-VBCHAM65)-D3 is illustrated in Figure 2.

**Table 1:** Polymer samples that have been tested in terms of antibacterial effect.

Polymer Sample	Code	Polymer Type	% Composition of Bacteriostatic Group	References
PSSAmC_16_	D1	Homopolymer of cetyltrimethylammonium 4-styrene sulfonate	100% QAC electrostatically attached	(Oikonomou *et al.*, 2012) [18]
PVBCHAM	D2	Homopolymer of vinyl benzyl dimethylhexadecylammonium chloride	100% QAC covalently attached	(Koromilas *et al.*, 2014) [22]
P(SSAmC_16_-co-VBCHAM65)	D3	Copolymer of cetyltrimethylammonium 4-styrene sulfonate and vinyl benzyl dimethylhexadecylammonium chloride	35% QAC electrostatically attached-65% QAC covalently attached	Present study
P(SSAmC_16_-co-VBCHAM25)	D3a	Copolymer of cetyltrimethylammonium 4-styrene sulfonate and vinyl benzyl dimethylhexadecylammonium chloride	75% QAC electrostatically attached-25% QAC covalently attached	Present study
PSSPhC_16_	D4	Homopolymer of hexadecyltributylphosphonium 4-styrene sulfonate	100% QPC electrostatically attached	(Oikonomou *et al.*, 2012) [18]
P(MMA-co-VBCHAM47)	D5	Copolymer of methyl methacrylate and vinyl benzyl dimethylhexadecylammonium chloride	47% QAC covalently attached	(Koromilas *et al.*, 2014) [22]
P(SSNa-co-VBCHAM20)	D6	Copolymer of sodium 4-styrene sulfonate and vinyl benzyl dimethylhexadecylammonium chloride	20% QAC covalently attached	(Koromilas *et al.*, 2014) [22]
P(SSNa-co-VBCHAM85)	D6a	Copolymer of sodium 4-styrene sulfonate and vinyl benzyl dimethylhexadecylammonium chloride	85% QAC covalently attached	(Koromilas *et al.*, 2014) [22]
P(AA-co-VBCHAM88)	D7	Copolymer of acrylic acid and vinyl benzyl dimethylhexadecylammonium chloride	88% QAC covalently attached	(Koromilas *et al.*, 2014) [26]
P(AA-co-VBCHAM20)	D7a	Copolymer of acrylic acid and vinyl benzyl dimethylhexadecylammonium chloride	20% QAC covalently attached	(Koromilas *et al.*, 2014) [26]

The success of polymerization was confirmed from the broad peaks at 6.2–7.7 ppm (3′, c′, 3, c), corresponding to the protons linked with the aromatic rings of both structural units. In addition, practically no monomer-related peaks were observed in the 5.5–6.5 ppm range, an indication that the monomers SSAmC_16_ or VBCHAM are no longer present but have been successfully polymerized. The presence of VBCHAM was also confirmed from the broad peak at 4.8 ppm (d) corresponding to the protons linked with the quaternary nitrogen atom (CH_2_N^+^). Moreover, the protons of the CH_3_ groups (8, i) were found at 0.86 ppm, whereas the thirteen CH_2_ groups (7, h) were identified at 1.28 ppm. The remaining CH_2_ groups (6, g) as well as the protons of the main chain appeared between 1.6 and 1.9 ppm. The peaks observed at 3.06 and 3.3 ppm were attributed to CH_3_ and CH_2_ groups (4, 5, e, f) linked with the nitrogen atoms. By ^1^Η-NMR quantification, from the integration of CH_2_N^+^ at 4.8 ppm and aromatic peaks at 6.2–7.7 ppm, it is concluded that the percentage of VBCHAM units in the copolymer is 65%. For all other preparations, the respective copolymers are summarized in Table 1. It is worth mentioning that a good agreement of the feed composition with the results estimated from the ^1^H-NMR spectra was observed in all cases. Finally copolymers composed from VBCHAM and acrylic acid units P(AA-co-VBCHAM) have been synthesized by free radical polymerization of the respective monomers. The absence of monomers was confirmed by ^1^Η-NMR, while the solubility of this product in water verifies that it is indeed a copolymer and not a mixture of two homopolymers. In fact, while the homopolymer poly(acrylic acid) is readily soluble in water and the homopolymer PVBCHAM-D2 is insoluble in water, the product P(AA-co-VBCHAM) is soluble in water forming a very viscous solution, as a consequence of its amphiphilic character.

### 2.2. Antibacterial Activity Studies

**Figure 3:**
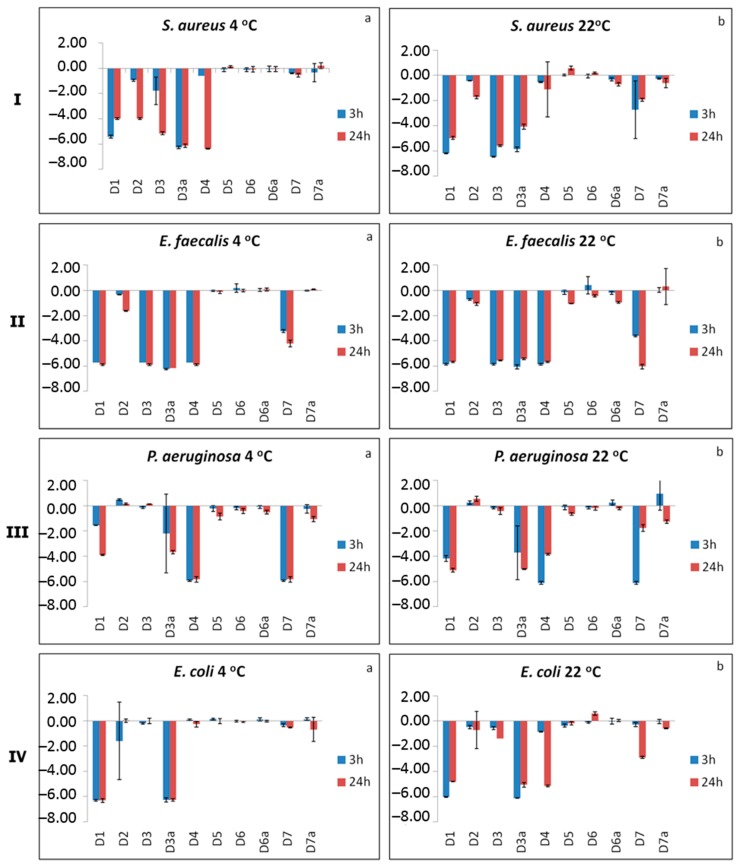
Bacteriostatic effect of polymers after 3 h and 24 h of contact with (**I**) *S. aureus*; (**II**) *E. faecalis*; (**III**) *P. aeruginosa* and (**IV**) *E. coli* at temperature 4 °C (**a**) and 22 °C (**b**). Each bar represents the log reduction from three independent experiments done in duplicates (mean ± standard deviation).

The antibacterial effect of the polymeric materials was evaluated against Gram-negative (*P. aeruginosa* and *E. coli*) and Gram-positive (*S. aureus* and *E. faecalis*) bacteria at different contact times (3 and 24 h) and temperature values (4 and 22 °C). The temperatures used in the study are based on the expected temperatures according to the proposed polymer use. The aim of the current study is to investigate the survival of bacteria on a wide range of polymers that might be suitable for use on surfaces for several applications and environments. The work involves the application of high bacterial cell concentrations (to represent a worst case scenario) onto each polymer and monitoring the bacterial levels over time at two temperatures: 20 °C and 4 °C, representing room and refrigerated temperature environments. Bacterial survival is extended at the lower, refrigeration temperature (4–8 °C) compared to room temperature (20 °C) in all cases. Extended survival times at the lower temperature were expected based on knowledge of bacterial physiology in environmental conditions. The results are presented in Figure 3I–IV, where log reduction values for each material were measured.

As observed the homopolymer PSSAmC_16_-D1 (bearing electrostatically attached quaternary ammonium groups) showed a high bacteriostatic effect against all the tested microorganisms. Specifically, a log reduction in the 4.0–6.5 range was detected after 3 and 24 h of contact at both temperatures. Only in the case of P. aeruginosa at 4 °C after 3 h of contact, a lower bacteriostatic activity was observed (1.5 log reduction) (Figure 3III-a). Moreover, the homopolymer PSSPhC_16_-D4, bearing electrostatically attached phosphonium groups, was also tested in terms of bacteriostatic activity. In fact, log reduction values between 5.7 and 6.1 were obtained for P. aeruginosa (Figure 3III) and *E. faecalis* (Figure 3II), under all experimental conditions. Moreover, a log reduction of 6.4 (4 °C) and 5.1 (22 °C) was observed for *S. aureus* (Figure 3I-a) and *E. coli* (Figure 3IV-b) respectively, after 24 h of contact. The results are in agreement with other published studies, where polymers with phosphonium salts exhibit strong antibacterial activity [27,28]. The homopolymer PVBCHAM-D2 (with covalently attached quaternary ammonium groups) showed a slight bacteriostatic effect at Gram-positive (*S. aureus* and *E. faecalis*) bacteria, with the greatest value (3.9 log reduction) observed after 24 h of contact at 4 °C for *S. aureus* (Figure 3I-a). On the other hand, no bacteriostatic effect appeared for the Gram-negative (*P. aeruginosa* and *E. coli*) bacteria. In some cases, 3 h efficacy is better than 24 h efficacy as observed, although this needs to be verified in more experiments.

Combining now the hydrophobic nature of methyl methacrylate with the covalently attached bacteriostatic group VBCHAM in the case of the copolymer P(MMA-co-VBCHAM47)-D5, a slight bacteriostatic effect only on *P. aeruginosa* at both temperatures, 4 °C and 22 °C, was observed (0.8–0.9 log reduction, Figure 3III). This effect is higher than that of the respective homopolymer PVBCHAM-D2. Moreover, a slight bacteriostatic effect was also observed on *E. faecalis* with the greatest value (1.1 log reduction) observed after 24 h of contact at 22 °C, but lower than that observed with PVBCHAM-D2. This contradictory behavior could not lead to a clear conclusion whether the presence of methyl methacrylate, assists the inhibition of the biofilm formation [29].

In a further attempt we tested the bacteriostatic effect of copolymers combining the hydrophilic monomer SSNa (the precursor of SSAmC_16_) with VBCHAM (providing covalent attachment of the bacteriostatic group) at various compositions. The SSNa-rich copolymer P(SSNa-co-VBCHAM20)-D6 did not demonstrate any remarkable bacteriostatic effect for the tested microorganisms, whereas the VBCHAM-rich copolymer P(SSNa-co-VBCHAM85)-D6a showed a slight bacteriostatic activity (0.7 and 1.0 log reduction) for *S. aureus* and *E. faecalis*, after 24 h of contact at 22 °C (Figure 3I-b,II-b, respectively). At lower temperature (4 °C), its efficiency was decreased. However, these trends were not repeated when SSNa was replaced by AA, although this monomer is also hydrophilic. In fact, the VBCHAM-rich copolymer P(AA-co-VBCHAM88)-D7 (with 88% bacteriostatic polymer) presented a high bacteriostatic activity against *E. faecalis* and *P. aeruginosa* (3.2–6.3 and 2.5–6.1 log reduction, respectively) at both contact times and temperatures (Figure 3II,III). Moreover, a bacteriostatic activity of 2.7 and 2.9 log reduction was observed against *S. aureus* (at 3 h of contact) and *E. coli* (at 24 h of contact), respectively, at 22 °C (Figure 3I-b,IV-b). The bacteriostatic activity of the AA-rich copolymer P(AA-co-VBCHAM20)-D7a was obviously lower than that of D7, in agreement with the lower bacteriostatic polymer content (20% in D7a *vs.* 88% in D7). In particular, a log reduction of 0.6 (22 °C), 1.1 (4 °C) and 0.6 (22 °C) was observed against S. aureus, *P. aeruginosa* and *E. coli*, respectively, after 24 h of contact (Figure 3I-b,III-a,IV-b). As it can be concluded, depending on the percentage of the ammonium groups bound to the copolymer, the antibacterial effect varies, and it may be affected by external factors, such as temperature.

Finally, the bacteriostatic effect of the new polymeric materials, bearing both covalently attached and electrostatically bound bacteriostatic units, was examined. In particular, the copolymer P(SSAmC_16_-co-VBCHAM65)-D3 inhibited to a great extent the growth of the Gram-positive (*S. aureus* and *E. faecalis*) bacteria presenting a log reduction between 5.5 and 6.5 at both contact times and temperatures tested (Figure 3I,II). Only against *S. aureus* the log reduction was rather low (1.8) after 3 h of contact at 4 °C. No bacteriostatic effect was observed for *E. coli* and *P. aeruginosa*, except for *E. coli* at 24 h of contact (22 °C) that there was a slight log reduction (1.4). In the case of P(SSAmC_16_-co-VBCHAM25)-D3a, a high bacteriostatic activity was observed against all the tested microorganisms (4–6.2 log reduction). The lower values were observed against *P. aeruginosa*, especially at 4 °C. The generally lower efficacy of the copolymers D3 and D3a against *P. aeruginosa* is similar to the antimicrobial effect of the homopolymer PSSAmC_16_-D1. This behavior may be attributed to the different membrane structures of Gram-positive and Gram-negative bacteria and the thickness of the peptidoglycan layer. This layer is thick in the case of Gram-positive bacteria (20–80 nm) and considerably thinner in the case of Gram-negative bacteria (7–8 nm) [30]. Accordingly, the different membrane structures may lead to changes in the permeability and the structural integrity of the membranes affecting the antimicrobial activity.

### 2.3. Antimicrobial Activity of Bacteriostatic Polysulfone Membranes

The antimicrobial efficiency of the pure novel polymeric biocides was explored in the previous section. However, in practical applications this activity should be maintained when the bacteriostatics are incorporated in solid matrices/membranes or when they are applied as coatings of surfaces. For example, a typical polymer often used in medical devices is polysulfone (PSF). Thus, as a final step of the present investigation, blends of PSF with the copolymers P(AA-co-VBCHAM88)-D7 and P(SSAmC_16_-co-VBCHAM25)-D3a, as well as with the homopolymer PSSAmC_16_-D1, were prepared and studied in respect to their bacteriostatic activity. The biocide content of the PSF membranes was 1%, 3% and 5%wt.

**Figure 4:**
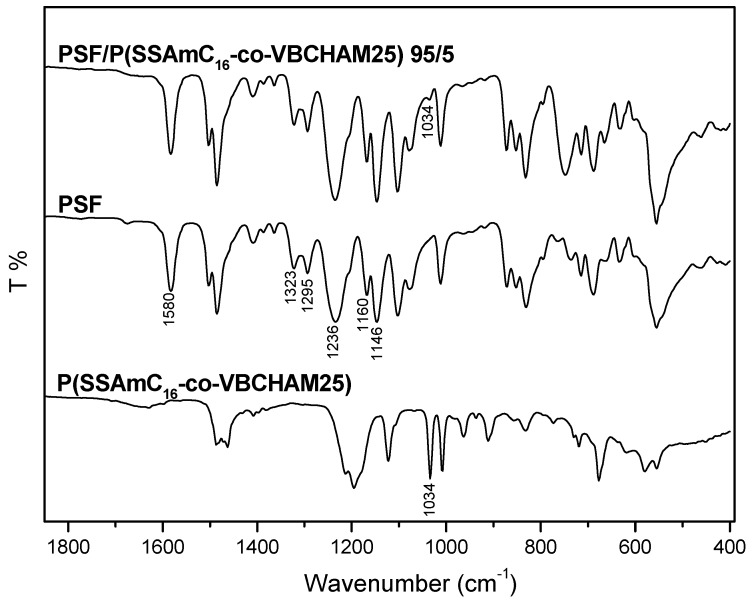
ATR-FTIR spectra of neat PSF, the copolymer P(SSAmC_16_-co-VBCHAM25)-D3a, as well as the membrane PSF/P(SSAmC_16_-co-VBCHAM25) at composition 95/5.

To verify the incorporation of the bacteriostatic materials in the PSF membranes, ATR characterization was first attempted. As an example, the ATR spectrum of the PSF membrane containing 5%wt P(SSAmC_16_-co-VBCHAM25)-D3a is compared in Figure 4 with the spectra of the two respective homopolymers, namely PSF and P(SSAmC_16_-co-VBCHAM25)-D3a. As expected, the PSF matrix can be easily identified in the blend, since several characteristic bands of PSF are easily observable [31,32]. More specifically, the characteristic absorption band at 1580 cm^−1^ and 1323 cm^−1^ are attributed to the C=C stretcing in the aromatic rings and the C-SO_2_-C assymetric streching, respectively. The peaks at 1295 cm^−1^ and 1236 cm^−1^ are assigned to the S=O asymetric stretching and the C-O-C symmetric stretching, respectively. Finally, the peaks at 1160 cm^−1^ and 1146 cm^−1^ are attributed to the C-SO_2_-C symetric streching and at the S=O symmetric stretching, respectively.

On the other hand, although slight changes in the ATR spectra of the final membrane are observed (*i.e.*, at 1034 cm^−1^, owed to the symmetrical vibration of SO_3_^−^ units), the presence of the copolymer P(SSAmC_16_-co-VBCHAM25)-D3a cannot easily be verified by this technique, probably due to its low content in the membrane.

**Figure 5:**
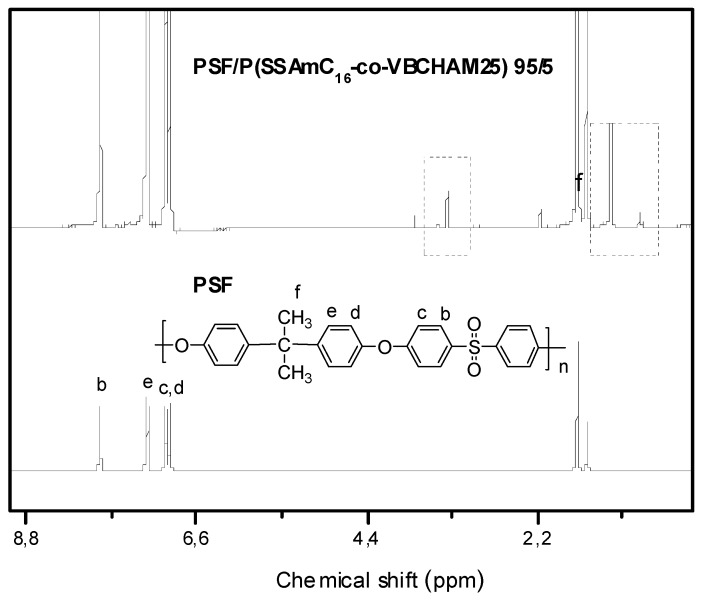
^1^H-NMR spectra of neat PSF, and the membrane PSF/P(SSAmC_16_-co-VBCHAM25) at composition 95/5 (%wt).

To further characterize the final bacteriostatic membranes we proceeded to ^1^H-NMR investigation. For this reason the membranes were dissolved in CDCl_3_ (a good solvent for both components). As an example, the ^1^H-NMR spectrum of the PSF membrane containing 5%wt of P(SSAmC_16_-co-VBCHAM25)-D3a is compared in Figure 5 with the spectra of neat PSF. In fact, apart form the peaks of PSF (methyl protons at 1.67 ppm and aromatic protons at 6.91–7.86 ppm), several characteristic peaks of the bacteriostatic polymer are now clearly detectable. Such peaks are seen in the areas 0.7–1.5 ppm and 2.9–3.6 ppm and they have been attributed in the discussion of Figure 2.

**Figure 6:**
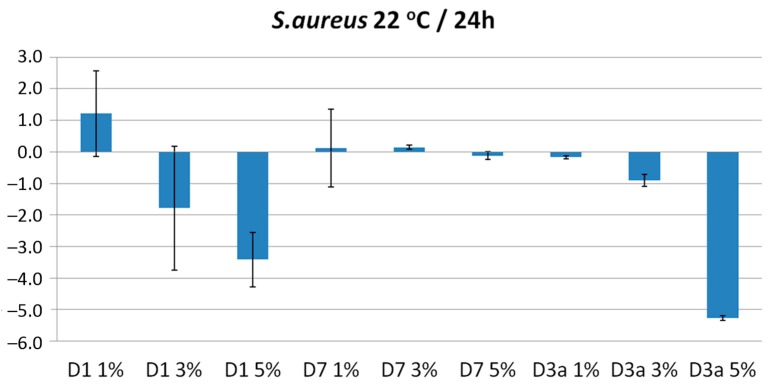
Bacteriostatic effect of polymer blends after 24 h of contact with *S. aureus* at temperature 22 °C. Each bar represents the log reduction from three independent experiments done in duplicates (mean ± standard deviation).

The bacteriostatic efficiency of the prepared blends was tested against *S. aureus*, which according to the data from the National Nosocomial Infection Surveillance (NNIS) study, is the most common pathogen found in surgical infections. The survival rate was tested at 24 h contact time and 22 °C. As seen in the results of Figure 6, no bacteriostatic activity was observed for the blends of PSF with the copolymer P(AA-co-VBCHAM88)-D7. On the contrary, a progressive increase of the bacteriostatic activity observed for the PSF membrane containing the copolymer D3a (bearing both covalently attached and electrostatically bound ammonium groups) with the highest value of 5.3 log reduction determined at a 5%wt bacteriostatic content.

The increasing bacteriostatic activity with the bacteriostatic content was also observed for the blends with PSSAmC_16_-D1, which showed a 3.5 log reduction at the highest D1 content. This value is lower than the one observed with the copolymer D3a, indicating that the combination of the covalent attachment with the electrostatic binding of the quaternary ammonium groups leads to enhanced bacteriostatic performance.

In general, from the abovementioned results it is realized that the chemical structure of the copolymer, the combination of the different components and parameters like temperature and contact time affected the survival of certain bacterial strains. In the case of temperature, both Gram-negative bacteria (*P. aeruginosa* and *E. coli*) demonstrated better survival at 4 °C, compared to 22 °C. On the other hand, Gram-positive bacteria (*S. aureus* and *E. faecalis*), demonstrated better survival at 22 °C, with the exception of PSSAmC_16_, which was more active at 22 °C against *S. aureus*. Wilks *et al.* [33] reported a difference in survival of *E. coli* O157 after contact with copper surfaces. According to their results, lower temperatures (4 °C), needed double time in order to reduce the bacterial number than room temperature, (20 °C). Our results are in compliance with this study, as *E. coli* was more resistant at 4 °C.

Contact time is also an important factor affecting the bacteriostatic activity of the polymers. This was shown in our study by testing two different contact times, 3 and 24 h. The increase of contact time yielded an increased bacteriostatic action in the cases of the homopolymers PSSAmC_16_-D1 with *P. aeruginosa*, PVBCHAM-D2 with *S. aureus* and *E. faecalis*, PSSPhC_16_-D4 with *E. coli* at 22 °C and *S. aureus* at 4 °C and the copolymer P(SSNaco-VBCHAM85)-D6a with *S. aureus* at 22 °C, *E. faecalis* at 22 °C and *P. aeruginosa* at 4 °C. However, the copolymers P(MMA-co-VBCHAM47)-D5, and P(SSNa-co-VBCHAM20)-D6 remained inactive even after 24 h of contact with the bacterial strains. In some cases, 3 h were sufficient enough to kill all the viable cells of the inoculum. This was observed with the homopolymers PSSAmC_16_ with *S. aureus*, *E. faecalis* and *E. coli* and PSSPhC_16_ with *E. faecalis* and *P. aeruginosa*. The high bacteriostatic efficiency of PSSAmC_16_ and PSSPhC_16_ makes them excellent candidates for replacing copper and the copper alloys in marine environment applications where anticorrosion properties are needed [27]. Generally, we may conclude that the polymers bearing either hydrophobic (MMA) or hydrophilic (SSNa) moieties in combination with the covalently attached bacteriostatic group VBCHAM did not show any substantial difference in terms of their antimicrobial properties, despite the fact that the homopolymer PVBCHAM presented bacteriostatic activity in some cases. However, when the hydrophilic monomer acrylic acid was used, instead of SSNa, for the copolymerization with VBCHAM, a different performance of the cells was detected. In particular, a pronounced bacteriostatic effect was observed not only at high (22 °C) but also at low temperature (4 °C), in most cases.

Another, remarkable result of the present study was the high antibacterial activity observed for the copolymers P(SSAmC_16_-co-VBCHAM65)-D3 and P(SSAmC_16_-co-VBCHAM25)-D3a, bearing ammonium groups not only covalently attached, but also electrostatically bound. More important, this antibacterial activity is maintained even when the copolymer D3a is incorporated in a PSF matrix. This behavior is very important for the future use of the copolymers in real applications. Bearing in mind that the copolymers could be applied onto selected substrates (surgical tools, ships’ bottoms, paints, *etc.*) by coating, spraying or roll transfer, the changing of various parameters, such as the polymer matrix and the blend composition, provide us the opportunity to control the release of the electrostatically bound ammonium group.

The method used, has been developed previously [33,34] for the bacteriostatic testing of copper surfaces. The testing method was adapted from the Environmental Protection Agency (EPA)- approved protocols. These protocols were prepared by ATS Laboratories (Eagan, MN, USA) for the International Copper Association [35]. In our study, the method has been proved efficient in testing antibacterial capability of polymers. Future experiments in this area could include toxicity tests or experiments for the antimicrobial activity of studied polymers to environmental strains of bacteria could be performed.

## 3. Experimental Section

### 3.1. Materials

The monomers methyl methacrylate (MMA), sodium 4-styrene sulfonate (SSNa), acrylic acid (AA) and 4-vinyl benzyl chloride (VBC), the polymers poly(sodium 4-styrene sulfonate) (PSSNa) and polysulfone (PSF), the initiator azobisisobutyronitrile (AIBN), the amine *N,N*-dimethylhexadecylamine (HAM), the surfactants cetyltrimethylammonium bromide (CTAB) and hexadecyltributylphosphonium bromide (HTPB), as well as deuterated dimethylsulfoxide (*d*_6_-DMSO) and deuterated chloroform (CDCl_3_) were purchased from Sigma-Aldrich (St. Louis, MO, USA) and used as received. The solvents *N*,*N*-dimethylformamide (DMF), *N*,*N*-dimethylacetamide (DMAc), chloroform (CHCl_3_), dimethyl sulfoxide (DMSO), acetone and hexane were purchased from Fisher Scientific (Fisher Scientific, Pittsburgh, PA, USA) and used as received. Ultra-pure water was obtained by means of a SG apparatus water purification unit.

### 3.2. Polymers Containing Quaternary Ammonium or Phosphonium Groups

#### 3.2.1. Bacteriostatic Functionalization of the Homopolymers

The homopolymers poly(cetyltrimethylammonium 4-styrene sulfonate) (PSSAmC_16_) and poly(hexadecyltributylphosphonium 4-styrene sulfonate) (PSSPhC_16_) were synthesized through electrostatic interaction between PSSNa and the surfactants cetyltrimethylammonium bromide (CTAB) or hexadecyltributylphosphonium bromide (HTPB), respectively [18,36]. The quaternization of the homopolymer poly(vinyl benzyl chloride) (PVBC), with *N*,*N*-dimethylhexadecylamine (HAM), took place after the polymerization of vinyl benzyl chloride through free radical polymerization (FRP) in CHCl_3_ using AIBN as initiator. The quaternized poly(vinyl benzyldimethyl-hexadecylammonium chloride) (PVBCHAM) bears ammonium groups attached covalently on the polymeric chain [22]. The chemical structures of the above mentioned quaternized homopolymers are shown in Figure 1.

#### 3.2.2. Synthesis of Pre- or Post-Polymerization Quaternization Materials

The copolymers methyl methacrylate-vinyl benzyl chloride (P(MMA-co-VBC)), sodium 4-styrene sulfonate-vinyl benzyl chloride (P(SSNa-co-VBC)) and acrylic acid-vinyl benzyl chloride (P(AA-co-VBC)) were synthesized through free radical polymerization in DMF, DMF/H_2_O and CHCl_3_, respectively using AIBN as initiator. The experimental procedures are described in details elsewhere [22,26]. All the copolymers were followed by the quaternization of VBC units with *N*,*N*-dimethylhexadecylamine (HAM). These copolymers will be denoted as P(MMA-co-VBCHAMx), P(SSNa-co-VBCHAMx) and P(AA-co-VBCHAMx), where x is the mol fraction of VBCHAM units in the copolymer, as determined by the ^1^H-NMR characterization. The chemical structures of the above mentioned copolymers are shown in Figure 1.

The copolymers cetyltrimethylammonium 4-styrene sulfonate-vinyl benzyl dimethylhexadecylammonium chloride (P(SSAmC_16_-co-VBCHAM)) were also synthesized through FRP. The two monomers were functionalized prior to the polymerization. In a next step, the desired quantity of the two functionalized monomers (total monomer concentration 1 M) were placed in a 100 mL round bottom flask, equipped with a reflux condenser, and dissolved in DMF. The solution was degassed, and the initiator AIBN (0.02 mol % over the total monomer concentration) was added. The reaction was left to proceed overnight under vigorous stirring in an Ar atmosphere in an oil bath set at 80 °C. After cooling down to room temperature, the copolymer was recovered by precipitation in ethyl acetate, filtered washed and dried in a vacuum oven at 80 °C for 24 h. The polymerization is described in Scheme 1. The characterization of the synthesized copolymers was conducted using ¹H-NMR in DMSO-*d*_6_ and ATR-FTIR spectroscopy.

### 3.3. Membrane Preparation

Blends of polysulfone with the homopolymer, PSSAmC_16_ (D1), as well as the copolymers P(SSAmC_16_-co-VBCHAM25) (D3a) and P(AA-co-VBCHAM88) (D7) were prepared via the wet casting phase inversion method. The polymer casting solution was prepared using DMAc as solvent at 80 °C under constant stirring until a homogeneous solution was obtained. The weight ratio of PSF/D1, D3 and D7 were: 99/1, 97/3, and 95/5, to form a 6 wt % solution. The casting solution, after dissolution, was poured onto a glass plate and placed in an oven at 80 °C, until total evaporation of the solvent. The prepared films were dried for 2 d under high vacuum at 80 °C in order to remove residual solvent.

### 3.4. Bacterial Culture Preparation

Certified reference materials (CRMs) were supplied from HPA (Porton Down, Salisbury, UK) in a LENTICULE^®^ disc format and were stored at −20 °C ± 5 °C. Bacterial strains used were: *E. coli* NCTC 9001, *S. aureus* NCTC 6571, *P. aeruginosa* NCTC 10662 and *E. faecalis* NCTC 775. To set up the stock culture, lenticules were added to 10 mL of peptone saline (Sodium chloride 8.5 g/L, Casein peptone 1.0 g/L) and left to rehydrate for 15–20 min. Each resulting bacterial culture was inoculated in 40 mL of Tryptic Soy Broth (TSB; Merck KGaA, Darmstadt, Germany) and incubated at 37 ± 1 °C for 18–24 h in order to yield ~10^8^ colony-forming units (cfu)/mL.

### 3.5. Preparation of Polymeric Samples for Bacteriostatic Testing

The polymeric compounds tested for their antimicrobial activity are shown in Table 1. The samples were homopolymers and copolymers that have been synthesized through free radical polymerization (FRP) [22]. The molar composition of the copolymers, as presented in Table 1, has been determined by ^1^H-NMR. Under aseptic techniques a polymer thin film of each sample was produced on a coupon cover glass 18 × 18 mm. The coupons were then transferred to a sterile Petri dish and stored at 25 °C prior to inoculation to prevent contamination.

### 3.6. Bacterial Reduction on Polymer Coupons

The method used was described previously [33,34]. Briefly, a 20 μL aliquot of an overnight culture of each bacterium was placed on every coupon coated with the polymer thin film. Prior to use, neat coupons were wiped with ethanol and allowed to air dry. They were also housed within a plastic container to minimize contamination from the laboratory environment. The polymer-coated coupons were stored in sterile petri dishes and incubated at two temperatures (22 °C and 4 °C) for two time periods (3 and 24 h). After the incubation, each coupon was transferred into a sterile 50 mL centrifuge tube containing 10 mL of sterile phosphate-buffered saline (PBS; Gibco^®^, London, UK) and 10–20 sterile 2 mm diameter glass beads (Merck KGaA, Darmstadt, Germany). Following vortex for 1 min, four serial decimal dilutions were performed using PBS. Nutrient agar plates (Merck KGaA, Darmstadt, Germany) were inoculated with 100 μL of each decimal dilution, which was spread over the surface of the agar with a sterile, glass spreader. Plates were incubated at 37 ± 1 °C for 18–24 h and enumerations of the colonies were recorded. For each dilution, two plates were inoculated and the result was expressed as the mean of the two numbers. In each experiment, cover glasses without polymer coating were used as control coupons, and also two coupons without any inoculated microorganism were tested, in order to test sterility. All experiments were replicated at least three times, by repeating on different days with different bacterial cell suspensions. Within each experiment several dilutions were made. The mean was calculated from a minimum of three data points. The detection limit for this experimental method was 100 bacteria based on counts of colony-forming units. As a negative control a coupon without a polymer was used. As a positive control, the bacterial broth on a coupon without the polymer was used. All the results of the experiments were estimated as a comparison in survival between the positive control and the survival on the polymer. The bacterial removal from coupon surfaces using the glass beads/vortex method was tested according previously reported method [33,34].

### 3.7. Statistical Analysis

Data was reported as the mean and standard error of the mean (SEM). Data was analyzed by statistical procedures (ANOVA) using IBM SPSS 21.0 (Armonk, NY, USA) to determine differences at the 5% significance level and graphical analyses were performed with Microsoft Excel (Microsoft, Redmond, WA, USA).

## 4. Conclusions

In the present study, novel polymeric biocides were developed and evaluated, concerning their antimicrobial activity on cultures of Gram-negative (*P. aeruginosa* and *E. coli*) and Gram-positive (*S. aureus* and *E. faecalis*) bacteria, as a function of contact time and temperature. These polymers were based on covalently attached and/or electrostatically bound quaternary ammonium bacteriostatic species. Overall, the present results reveal the decisive effect of the chemical structure on the bacteriostatic performance of the novel polymeric materials. For example, the use of AA as co-monomer, instead of SSNa, leads to efficient antimicrobial polymers, based on covalently attached quaternary ammonium groups. In addition, the combination of covalently attached with electrostatically bound quaternary ammonium groups on the same polymer chain provides remarkably effective polymeric biocides, even when these materials are incorporated in PSF matrices.

The results of this study may contribute to the design of novel antimicrobial materials as well as an understanding of the influence of the chemical structure on the bacteriostatic activity. Furthermore, these materials are promising candidates for a range of applications, covering food packaging, medical applications or even marine applications. Based on the results reported in this paper, the copolymeric structures exhibiting the best antimicrobial efficiency have already been selected for the synthesis of copolymers with more complex architectures, like block or graft polymers, in order to combine the antimicrobial properties with the control in release of the active groups, due to their ability to self-organize in different polymeric matrixes. The results from this parallel study will be reported in future works.
